# Cyclooxygenase-2 Suppresses the Anabolic Response to PTH Infusion in Mice

**DOI:** 10.1371/journal.pone.0120164

**Published:** 2015-03-17

**Authors:** Shilpa Choudhary, Ernesto Canalis, Thomas Estus, Douglas Adams, Carol Pilbeam

**Affiliations:** 1 New England Musculoskeletal Institute, University of Connecticut Health Center, Farmington, Connecticut, United States of America; 2 Department of Medicine, University of Connecticut Health Center, Farmington, Connecticut, United States of America; Inserm U606 and University Paris Diderot, FRANCE

## Abstract

We previously reported that the ability of continuously elevated PTH to stimulate osteoblastic differentiation in bone marrow stromal cell cultures was abrogated by an osteoclastic factor secreted in response to cyclooxygenase-2 (Cox2)-produced prostaglandin E_2_. We now examine the impact of *Cox2* (*Ptgs2*) knockout (KO) on the anabolic response to continuously elevated PTH *in vivo*. PTH (40 μg/kg/d) or vehicle was infused for 12 or 21 days in 3-mo-old male wild type (WT) and KO mice in the outbred CD-1 background. Changes in bone phenotype were assessed by bone mineral density (BMD), μCT and histomorphometry. PTH infusion for both 12 and 21 days increased femoral BMD in *Cox2* KO mice and decreased BMD in WT mice. Femoral and vertebral trabecular bone volume fractions were increased in KO mice, but not in WT mice, by PTH infusion. In the femoral diaphysis, PTH infusion increased cortical area in *Cox2* KO, but not WT, femurs. PTH infusion markedly increased trabecular bone formation rate in the femur, serum markers of bone formation, and expression of bone formation-related genes, growth factors, and Wnt target genes in KO mice relative to WT mice, and decreased gene expression of Wnt antagonists only in KO mice. In contrast to the differential effects of PTH on anabolic factors in WT and KO mice, PTH infusion increased serum markers of resorption, expression of resorption-related genes, and the percent bone surface covered by osteoclasts similarly in both WT and KO mice. We conclude that Cox2 inhibits the anabolic, but not the catabolic, effects of continuous PTH. These data suggest that the bone loss with continuously infused PTH in mice is due largely to suppression of bone formation and that this suppression is mediated by Cox2.

## Introduction

Parathyroid hormone (PTH) is a major systemic regulator of calcium homeostasis and bone turnover. PTH acts on a G-protein coupled receptor, PTH1R, expressed on osteoblast lineage cells to stimulate bone formation via Gαs/cAMP-activated pathways [1–4]. Intermittent PTH was the first anabolic agent approved for osteoporosis therapy in the USA [5,6]. When PTH is injected intermittently, both bone formation and resorption are increased but formation is increased more than resorption. On the other hand, when PTH levels are continuously elevated, resorption is greater than formation and bone is lost [7,8]. It is unclear if this change in the bone turnover from net formation to net resorption is due to increased resorption with continuous PTH, compared to intermittent PTH, or if it is the formation response that is reduced. A number of *in vitro* studies report that continuous PTH treatment inhibits osteoblast differentiation [3,9–11], and some *in vivo* studies conclude that continuous PTH infusion suppresses bone formation [12]. These observations suggest that the bone loss associated with continuous PTH infusion is not simply the result of increased resorption but may involve suppressed bone formation.

PTH is also a potent inducer of cyclooxygenase-2 (Cox2), the major enzyme involved in prostaglandin (PG) production [13]. PGs are locally produced lipids that are made by, and act on, both osteoblasts and hematopoietic cells [13–16]. PGE_2_ is abundantly expressed in bone, and similar to PTH, PGE_2_ can stimulate both bone resorption and formation via Gαs/cAMP-activated pathways [17]. Injected PGE_2_ increases both bone resorption and formation, but formation can be greater than resorption and increase bone mass in rats, dogs and humans [13,18,19]. Similar to PTH, continuous PGE_2_ administration in rats can lead to bone loss, whereas intermittent administration is anabolic [20].

Because of their similar actions, we initially proposed that PTH-induced Cox2 expression and PGE_2_ might mediate some of the anabolic effects of PTH on bone. Instead, we found that intermittent PTH *in vivo* was more anabolic in *Cox2* knockout (KO) mice than in wild type (WT) mice, suggesting that PGE_2_ suppressed the anabolic effects of PTH *in viv*o [21]. *In vitro*, in bone marrow stromal cell (BMSC) cultures, continuous PTH could markedly stimulate osteoblast differentiation when either (1) Cox2 produced PGE_2_ was absent or (2) osteoclast formation was prevented by adding osteoprotegerin (Opg) to prevent receptor activator of nuclear factor κB ligand (Rankl) from binding to its receptor (Rank) on hematopoietic cells [22]. We went on to show that, in the presence of Cox2 or PGE_2_, murine bone marrow macrophages stimulated by Rankl to commit to the osteoclast lineage secreted a factor that could be transferred in the medium to block PTH-stimulated differentiation in primary osteoblast cultures [23]. The production of this inhibitory factor may explain why it has been difficult to demonstrate PTH stimulation of osteoblast differentiation *in vitro* except when cells had brief, transient exposure to PTH [9,11,24]. Transient exposure studies generally remove PTH-containing media, replacing with fresh media, a procedure that should also remove PGE_2_ that accumulates in the media.

The *in vivo* model most likely to reproduce our *in vitro* effects is the continuous infusion model. PTH is rapidly metabolized *in vivo*, *Cox2* is a transiently inducible gene, and PGs are rapidly released from cells and degraded as they transit the lungs [13]. Thus, the intermittent or daily injection PTH protocol is expected to result in very brief periods of jointly elevated PTH and Cox2/PGE_2_. In the continuous infusion protocol, it is likely that the elevation of Cox2/PGE_2_ is sustained and the interaction of PTH and PGE_2_ becomes more important. This current study was undertaken to test the hypothesis that, similar to the osteogenic response *in vitro*, the anabolic response *in vivo* to continuous PTH elevation would be increased in the absence of *Cox2*.

## Materials and Methods

### Materials

ALZET micro-osmotic pumps (model 1004D) were from Durect Corp. (Cupertino, CA). Human parathyroid hormone (hPTH; 1–34; H-4835) was from Bachem Bioscience Inc. (Torrance, CA). All other chemicals were from Sigma Aldrich (St. Louis, MO), unless otherwise noted.

### Animals

Mice with disruption of *Ptgs2*, which produce no functional Cox2 protein, called *Cox2* KO mice, in a C57BL/6,129SvJ background were the gift of Scott Morham [25]. Because *Cox2* KO mice in these inbred backgrounds have renal failure and females are infertile [26], we backcrossed these mice 20 generations into the outbred CD-1 background. In the outbred CD-1 background, *Cox2* KO mice do not develop renal failure and females are fertile [21]. Mice were genotyped as described previously [21]. All animal studies were conducted in accordance with IACUC protocol 100590–0316, “COX-2 Regulation of Bone Responses to PTH,” approved by the Animal Care and Use Committee of the University of Connecticut Health Center.

Experimental mice were bred by crossing WT with WT and *Cox2* KO with KO mice all in the outbred CD-1 background. This breeding protocol markedly reduces the number of mice needed because heterozygotes, which are not used for experiments, are not produced and also reduces experimental costs associated with genotyping, as well as discomfort to the mice. Because maintenance of separate colonies of WT and KO mice could lead to genetic drift that might affect phenotype, we restart or “refresh” maintenance colonies twice a year by mating *Cox2* KO mice with WT mice purchased from Jackson Laboratory (Bar Harbor, ME). The heterozygotes produced are mated to produce new WT and KO mice that are then used to establish new breeding colonies for experimental mice.

### Continuous PTH infusion protocol

In the first study, 3-mo-old male WT and *Cox2* KO mice (n = 7–8 mice/group) were infused with vehicle or PTH (40 μg/kg/d) for 12 d. In the second study, 3-mo-old male WT and *Cox2* KO mice (n = 6–7 mice/group) were infused with vehicle or PTH (40 μg/kg/d) for 21 d. For both studies, ALZET micro-osmotic pumps (model 1004D with flow rate of 0.11 μl/h and delivery time of 4 weeks) filled with 100 μl of vehicle (0.001 N hydrochloric acid-acidified 0.1% BSA in 1x phosphate buffered saline, PBS) or PTH were surgically implanted under isoflurane (1.5–2% with 250 ml/min O_2_) anesthesia into the subcutaneous cavity of the mid-scapular region of mice following the manufacturer’s instructions www.alzet.com. The filled pumps were primed under sterile conditions in saline at 37° C for 6 h prior to implantation. After implantation, mice were checked regularly for any signs of distress and weighed every 3^rd^ day.

### Serum measurements

Blood was obtained by heart puncture at the end of each experiment after euthanasia with gaseous carbon dioxide. Blood was allowed to clot at room temperature, and serum collected after centrifugation of samples at 5000 rpm for 10 minutes. Serum from each animal was divided into aliquots and frozen at -80°C. Calcium was measured using a kit from Eagle Diagnostics (De Soto, TX) in accordance with the manufacturer’s instructions. N-terminal propeptide of type I procollagen (PINP) and C-terminal telopeptide (CTX) were assayed using the Rat/Mouse PINP and Mouse/RatLaps CTX EIA kits from IDS (Fountain Hills, AZ). Serum osteocalcin was measured using the Mouse Osteocalcin ELISA Kit from Immutopics (San Clemente, CA). Serum PGE_2_ was measured using ELISA Kit (K015-H1) from Arbor Assays (Ann Arbor, MI).

### *In vivo* Xray absorptiometry (DXA)

Femoral bone mineral density (BMD) was measured by PIXImus2 Densitometer (GE Medical Systems, Madison, WI) in mice anesthetized with isoflurane (1.5–2% isoflurane with 250 ml/min O_2_). *In vivo* BMD of the right femur was measured 1 day before the implantation of the pump and at the end of the study, and the change in BMD calculated as percent difference from baseline for each mouse. Percent body fat was also measured by DXA. The region of interest, which was set to exclude the head, was from the C3 cervical vertebra to the S3 sacral vertebra.

### *Ex vivo* μCT imaging

Right femurs and lumbar vertebrae L1–L6, dissected of connective tissue, were fixed in 70% ethanol (femurs from 21 day infusion, all vertebrae) or 10% buffered formalin (femurs from 12 day infusion). Trabecular morphometry within the metaphyseal region of the distal femur and center of the third lumbar vertebra (L3) and cortical morphometry at the femoral mid-diaphysis were quantified using conebeam micro-focus X-ray computed tomography (μCT40, ScanCo Medical AG, Bassersdorf, Switzerland). Serial tomographic images were acquired at 55 kV and 145 μA, collecting 1000 projections per rotation at 300 ms integration time. Three dimensional 16-bit grayscale images were reconstructed using standard convolution back-projection algorithms, rendering a 12 mm field of view at a discrete density of 578,704 voxels/mm^3^ (isometric 12 μm voxels). Segmentation of bone from marrow was performed in conjunction with constrained Gaussian filter, applying hydroxyapatite-equivalent density thresholds of 470 and 710 mg/cm^3^ for trabecular and cortical compartments, respectively. Volumetric regions of interest (ROI) for trabecular analysis in the femur were defined within endosteal borders. For trabecular morphometry in the distal femur, ROI was defined within the endosteal borders and was located 1 mm away from midline of growth plate extending 1 mm proximally. In the vertebral bodies, ROI included the central 80% of vertebral height. Trabecular morphometry included bone volume fraction (BV/TV), trabecular thickness (Tb.Th), trabecular number (Tb.N), trabecular spacing (Tb.Sp), connectivity density (Conn.Dens), and structural model index (SMI). The lower the SMI, the more likely trabeculae resemble plates rather than rods. Cortical measurements included cortical area (excluding intracortical porosity) and thickness, intracortical porosity, and subperiosteal and subendosteal areas.

### Static and dynamic histomorphometry

Mice were injected intraperitoneally with calcein (20 mg/kg) and demeclocycline (50 mg/kg), 7 and 2 days before sacrifice, respectively, for dynamic histomorphometry. Both static and dynamic measurements were done on the OsteoMeasure analysis system (Osteometrics, Atlanta, GA). Right femurs were used for static histomorphometry and left femurs were used for dynamic histomorphometry. The terminology and units used are those recommended by the Histomorphometry Nomenclature Committee of the ASBMR [27].

For static histomorphometry, femurs fixed in 10% buffered formalin were decalcified in 15% EDTA, dehydrated in ethanol, cleared in xylene and embedded in paraffin. Longitudinal sections, 5 μm thick, were cut on a microtome (Microm; Richards-Allan Scientific, Kalamazoo, MI), stained for tartrate resistant acid phosphatase (TRAP) and counterstained with hematoxylin before measurements. Static parameters were measured in a defined area between 200 and 2000 μm from the growth plate, encompassing an area of 2.08 mm^2^. Parameters included BV/TV, Tb.Th, Tb.N, Tb.Sp, osteoblast surface per bone surface (Ob.S/BS) and osteoclast surface per bone surface (Oc.S/BS).

For dynamic histomorphometry, 10 μm thick, longitudinal sections were cut on femurs fixed in 70% ethanol and embedded undecalcified in methyl methacrylate. Mineralizing surface per bone surface (MS/BS) and mineral apposition rate (MAR), calculated as the distance between the midpoints of double labels over the time interval, were measured on unstained sections under UV light, using a diamidino-2-phenylindole fluorescein isothiocyanate Texas red filter. The ROI for measurements was same as used for static analysis. Bone formation rate per bone surface (BFR/BS) was calculated as the product of MAR and MS/BS.

### Real-time (quantitative) PCR analysis

Both tibiae were excised at the end of the experiment and combined for RNA. Tibial ends were cut off but marrow was not flushed. Total RNA was extracted with Trizol (Invitrogen, Grand Island, NY) following the manufacturer’s instructions. Five μg of total RNA was DNase treated (Ambion, Inc., Austin, TX) and converted to cDNA by the High Capacity cDNA Archive Kit (Applied Biosystems, Foster City, CA). PCR was performed in 96-well plates. Both Assays-on-Demand Gene Expression Taqman primers (Applied Biosystems) and validated Syber Green primers (*http://pga.mgh.harvard.edu/primerbank*) were used for PCR (Table 1). *Glyceraldehyde-3-phosphate dehydrogenase* (*Gapdh*) or *β-actin* served as endogenous control. All primers were checked for equal efficiency over a range of target gene concentrations. Each sample was amplified in duplicate. PCR reaction mixture was run in Applied Biosystems Prism 7300 Sequence Detection System instrument utilizing universal thermal cycling parameters. Data analysis was done using relative quantification (RQ, ΔΔCt) or relative standard curve method.

**Table 1:** List of genes and primers used to analyze gene expression by quantitative real time PCR.

(A) Gene Name	Probe Number	Gene Name	Probe Number
*Acp5* (*Trap*)	Mm00475698_m1	*Bmp2*	Mm01340178_m1
*Ccl2* (*Mcp1*)	Mm00441242_m1	*cFos*	Mm00487425_m1
*Dkk1*	Mm00438422_m1	*Fgf2*	Mm00433287_m1
*Gapdh*	Mm99999915_g1	*Igf1*	Mm00439560_m1
*Il17a*	Mm00439618_m1	*Mepe*	Mm02525159_s1
*Ramp3*	Mm00840142_m1	*Runx2*	Mm00501584_m1
*Sfrp1*	Mm00489161_m1	*Sost*	Mm00470479_m1
*Tnfα*	Mm00443258_m1	*Tnfsf11*(*Rankl*)	Mm00441908_m1
*Tnfrsf11b* (*Opg*)	Mm01205928_m1	*Wisp1*(*Ccn4*)	Mm01200484_m1
*Wnt4*	Mm01194003_m1	*Wnt10b*	Mm00442104_m1
*Wnt3a*	Mm00437337_m1	*Ptgs2 (Cox2)*	Mm00478374_m1
**(B) Gene Name**		**Primer Sequence**	
*Bglap (Osteocalcin)*		Rev TGG TCT GAT AGC TCG TCA CAA G For CTG ACC TCA CAG ATC CCA AGC	
*Actin*		Rev CCA GTT GGT AAC AAT GCC ATG T For GGC TGT ATT CCC CTC CAT CG	

(A) Genes analyzed by TaqMan Gene Expression Assay and the Design Probes used to analyze their expression. (B) Genes analyzed by Syber Green validated primer sequences.

### Statistics

All data are presented as means ± SEM. Data analysis was performed using Sigma Plot 11.0 (Systat Software, Inc., Chicago, IL). To assess the effect of genotype on treatment response, data were analyzed by two-way ANOVA, followed by post hoc Bonferroni pairwise multiple comparisons. If effects of genotype and treatment were determined by two-way ANOVA to be independent, data were analyzed by one-way ANOVA, followed by post hoc Bonferroni pairwise multiple comparisons. If data were not normally distributed, they were log_10_^-^ transformed before performing two-way ANOVA, or if effects of genotype and treatment were independent, they were analyzed by one-way ANOVA on ranks, followed by Dunn’s Test for all pairwise multiple comparisons. In one instance, data could only be normalized when an outlier (different from group mean by >2 times the SD of the group before removal) was removed. Comparison of 2 variables was by t-test. Differences were considered significant if p < 0.05. Values of p < 0.01 were set to p < 0.01.

## Results

We used adult male mice at 3-months of age for studies to avoid the rapid skeletal growth phase that might make effects of PTH difficult to interpret. The dose and duration of PTH infusion were chosen based on earlier studies showing the catabolic effects of continuous PTH infusion [7,8].

### Basal phenotype differences

On comparison of body weights of 3-month old male WT and *Cox2* KO mice 1 day prior to starting infusion, pooled from both 12 and 21 day experiments, *Cox2* KO mice weighed 10% less than WT mice (Table 2). In our previous study of 6-mo-old male WT and *Cox2* KO mice in the same CD-1 background, KO mice weighed 9% less than WT mice [21]. WT and KO mice do not appear different in size, and femur lengths of vehicle-treated WT and KO mice, measured *ex vivo* at the end of infusion by μCT, were the same (Table 2). Percent body fat measured by DXA *in vivo* prior to starting treatments was 17% lower in KO mice than WT mice (Table 2). There was no difference in lean mass. We found similar results in our previous study of 6-month old WT and KO mice [21]. In that study, % body fat was 18% lower (and lean mass no different) in KO compared to WT mice (unpublished data). Hence, lower % body fat might contribute to the lower body weights of *Cox2* KO mice. Finally, by pooling the BMDs from WT and KO mice from both infusion experiments, measured 1 day prior to starting treatment, there was a small difference (5%) in BMD between WT and KO mice, which was also observed previously [21]. Because this difference is small, it may not be evident in smaller experimental groups.

**Table 2:** Comparison of wild type (WT) and *Cox2* KO mice.

Parameter	Number	WT mice	*Cox2* KO mice
Weight (g)	28	26.7 ± 0.19	24.0 ± 0.12^a^
BMD (mg/mm^2^)	28	65.6 ± 0.66	62.4 ± 0. 35^a^
Body fat (%)	15	15.7 ± 0.11	13.0 ± 0.10^a^
Lean mass (g)	15	20.5 ± 0.19	20.0 ± 0.12
Femur length (mm)	13	15.3 ± 0.05	15.3 ± 0.08

Mice from both 12-d and 21-d infusion studies were pooled for weight, BMD, and femur length. Lean mass and % fat were measured only in the 12-d study. Except for femur length, which was measured on vehicle-treated mice at the end of infusion, all other parameters were measured 1 day prior to beginning infusion. Data are means ± SEM for (n) mice.

^a^Significant effect of genotype, p<0.01. BMD, % fat and lean mass were measured *in vivo* by DXA. Femur lengths were measured *ex vivo* by μCT.

### Study 1: PTH infusion for 12 days

WT and *Cox2* KO mice did not appear to be in distress nor did they lose weight with infusion despite becoming hypercalcemic (Table 3). PTH infusion increased serum Ca similarly in WT and KO mice from 9.8 mg/dl to 11.9–12.1 mg/dl. To confirm that the induction of *Cox2* produced PGE_2_, we measured serum levels of PGE_2_. Although Cox1 is constitutively expressed [13,17,21,26], no serum PGE_2_ was detectable in *Cox2* KO mice. Serum PGE_2_ was detectable in 3 out of 6 samples in WT mice and was increased 25-fold by PTH (Table 3). Serum PGE_2_ is rapidly degraded in the circulation [13], and the levels measurable in serum are likely to be much lower than those seen locally by cells producing PGE_2_ in the bone environment.

**Table 3:** Body weight, serum calcium (Ca) and serum PGE_2_ in wild type (WT) and *Cox2* KO mice infused with vehicle or PTH for 12 days.

Parameter	WT mice		*Cox2* KO mice	
	Vehicle	PTH	Vehicle	PTH
Start weight (g)	27.3 ± 0.26 (7)	27.0 ± 0.63 (8)	24.1 ± 0.18^b^ (7)	24.2 ± 0.18^b^ (8)
End weight (g)	28.9 ± 0.32 (7)	28.4 ± 0.30 (8)	25.7 ± 0.18^b^ (7)	25.9 ± 0.18^b^ (8)
Ca (mg/dl) at end	9.8 ± 0.11 (7)	12.1 ± 0.17^a^ (8)	9.8 ± 0.07 (7)	11.9 ± 0.10^a^ (8)
PGE_2_ (pg/ml) at end	5.2 ± 1.37 (3)*	127.7 ± 13.80^a^ (6)	UD (6)	UD (6)

Data are means ± SEM for (n) mice.

^a^Significant effect of PTH, p<0.01.

^b^Significant effect of genotype, p<0.01.

*Three of the 6 samples were below the limit of detectability of the assay (3.25 pg/ml) and the mean was calculated from the remaining 3 samples. Hence, the value is an overestimate of true value. UD = undetectable. 352 pg/ml = 1 nM PGE_2_

**Fig 1:**
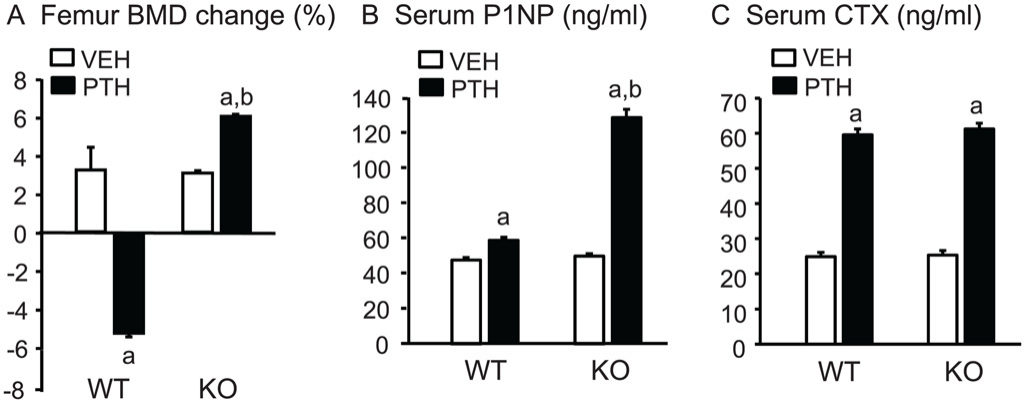
Femoral BMD and serum markers of bone turnover in mice infused for 12 days. **A.** BMD was measured *in vivo* at beginning and end of infusion with vehicle (VEH) or PTH (40 μg/kg/d) in WT and *Cox2* KO mice and the percent change calculated relative to the beginning BMD for each mouse. **B, C.** Serum parameters for bone formation (P1NP) and bone resorption (CTX) were measured at the time of euthanasia. Bars are means ± SEM for 7 WT and 7 KO mice treated with vehicle and 8 WT and 8 KO treated with PTH. ^a^Significant effect of PTH, p<0.01. ^b^Significant effect of genotype, p<0.01.

**Percent change in BMD and serum markers of turnover.** There was no significant difference between the mean femoral BMD of WT and KO mice at baseline (0.0647±0.0009 vs 0.0625 ±0.0004 g/cm^2^, respectively). The mice were still growing and the % BMD change was approximately 3% for both vehicle-infused WT and KO mice at the end of the experiment (Fig. 1A). The % change in femoral BMD by PTH infusion was doubled in KO mice and converted to a 5% decrease in WT mice (Fig. 1A). There was no significant difference in PINP, a serum marker of bone formation, or CTX, a serum marker of bone resorption, between vehicle-infused WT and *Cox2* KO mice at the end of infusion (Fig. 1B,C). PTH increased serum PINP to a greater extent in KO mice (150%) than in WT mice (20%). In contrast, PTH increased CTX similarly (2.4-fold) in both WT and *Cox2* KO mice (Fig. 1C).

**Fig 2:**
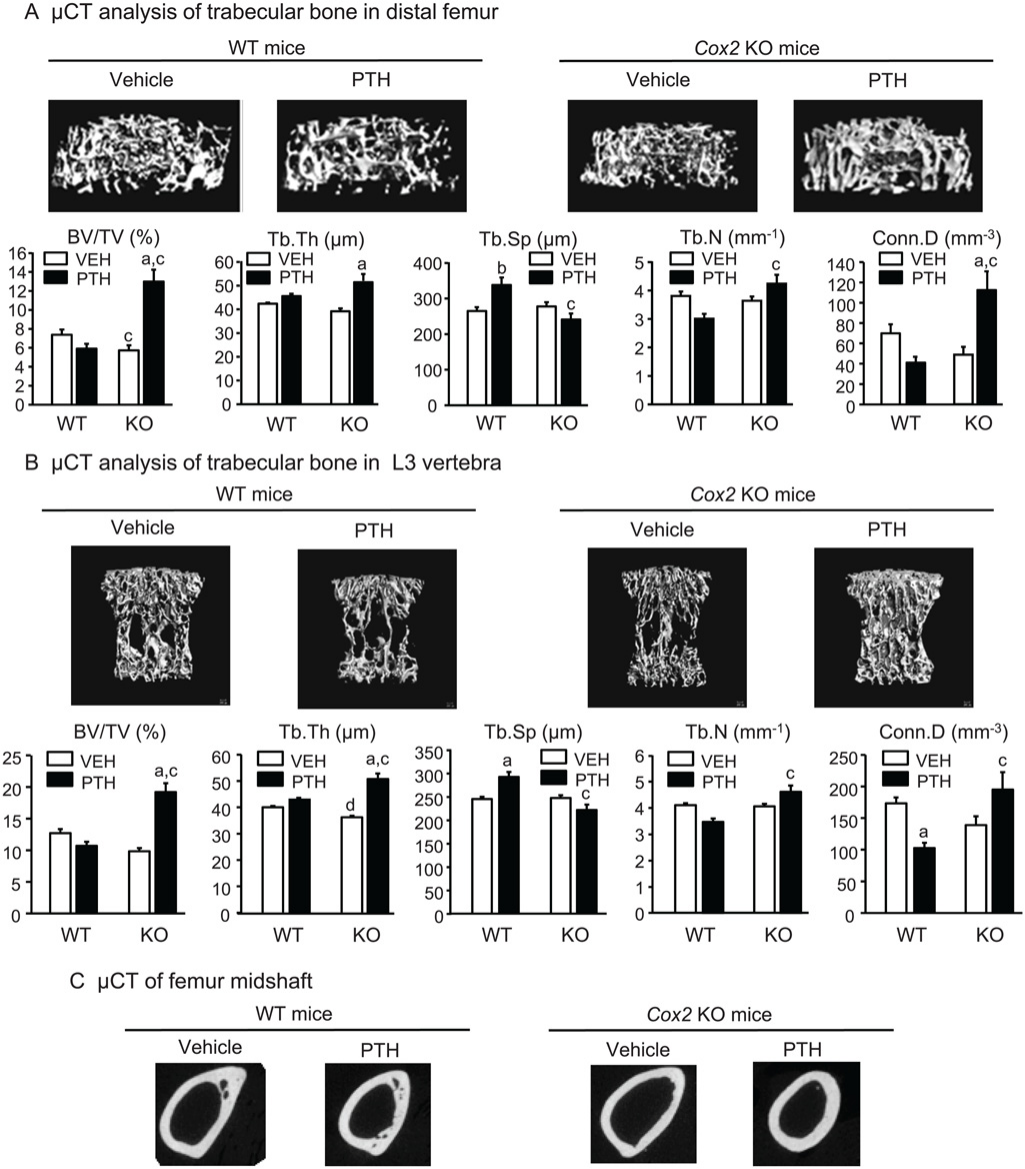
Morphometry of femoral bone and L3 vertebral bone in mice infused for 12 days. **A.** Representative μCT longitudinal images (top panel) and morphometric analyses (bottom panel) of trabecular bone in the metaphyseal region of distal femur. Bars are means ± SEM for 7 WT and 7 KO mice treated with vehicle and 8 WT and 8 KO mice treated with PTH. **B**. Representative μCT cross-sectional images (top panel) and morphometric analyses (bottom panel) of trabecular bone in the L3 vertebrae. Bars are means ± SEM for 7 WT and 7 KO mice treated with vehicle and 8 WT and 8 KO mice treated with PTH. **C**. Representative μCT cross-sectional images of midshaft of femurs. ^a^Significant effect of PTH, p<0.01; ^b^p<0.05. ^c^Significant effect of genotype, p<0.01; ^d^p<0.05.

**μCT analyses of femur and vertebra.** By μCT analysis of the distal femurs, there was a decrease in trabecular volume fraction (BV/TV) in vehicle-infused *Cox2* KO mice compared to WT mice (5.72±0.55% and 7.37±0.56%, respectively) (Fig. 2A). Compared to vehicle controls, PTH infusion increased BV/TV 113% in KO femurs. There was a non-significant decrease in BV/TV of 19% in WT femurs. This resulted in BV/TV being 2.1-fold greater in the PTH-infused KO group compared to the PTH-infused WT group. There were no significant differences between vehicle-infused WT and KO mice in femoral Tb.Th, Tb.Sp, Tb.N or trabecular connectivity (Conn.Dens) (Fig. 2A). PTH increased Tb.Th by 30% in KO femurs and increased Tb.N relative to WT femurs. Tb.Sp was increased in WT femurs by PTH infusion and decreased in KO femurs relative to WT. PTH infusion decreased Conn.Dens 59% in WT femurs (non-significant) and increased Conn.Dens 112% in KO femurs. SMI in vehicle-treated WT and KO femurs was 2.78± 0.07 and 2.82 ± 0.12, respectively, and in PTH-treated WT and KO mice, 2.74 ± 0.04 and 2.58 ± 0.04, respectively. The decrease in SMI with PTH in KO mice was not significant.

On μCT analysis of the L3 vertebrae (Fig. 2B), the only statistically significant difference between vehicle-treated controls was a small decrease in Tb.Th in KO mice compared to WT mice (40.0±0.55 and 36.2±0.62 μm, respectively). PTH increased vertebral BV/TV in KO mice 95% while there was a (non-significant) decrease in BV/TV of 16% in WT mice. In KO mice, PTH increased Tb.Th by 40% and increased Tb.N relative to WT. Tb.Sp was increased in WT mice by PTH infusion and decreased in KO mice relative to WT mice. PTH infusion decreased Conn.Dens 40% in WT mice and increased Conn.Dens 40% in KO mice. SMI in vehicle-treated WT and KO femurs was 1.94± 0.06 and 2.24 ± 0.09, respectively, and in PTH-treated WT and KO mice, 2.12 ± 0.05 and 1.47 ± 0.12, respectively. The decreases in SMI with PTH treatment in KO mice relative to vehicle and to PTH-treated WT mice were significant (p<0.01), suggesting that the trabeculae became more plate-like in the KO mice with PTH.

Cortical morphometry by μCT of WT and KO femurs at mid-diaphysis was similar between vehicle-infused WT and *Cox2* KO mice (Table 4, Fig. 2C). PTH infusion increased cortical area and cortical thickness by 25% in KO femurs but had no significant effect on WT cortical parameters.

**Table 4:** Cortical morphometry by μCT in the midshaft femoral region of WT and *Cox2* KO mice after infusion with vehicle or PTH for 12 days.

Parameter	WT mice		*Cox2* KO mice	
	Vehicle (7)	PTH (8)	Vehicle (7)	PTH (8)
Cortical area (mm^2^)	1.02 ± 0.03	0.97 ± 0.03	0.98 ± 0.02	1.23 ± 0.07^a^ ^,^ ^b^
Cortical thickness (mm)	0.21 ± 0.01	0.19 ± 0.01	0.20 ± 0.01	0.25 ± 0.01^a^ ^,^ ^b^
Cortical porosity (%)	0.55 ± 0.09	0.56 ± 0.07	0.57 ± 0.07	0.39 ± 0.03
Subperiosteal area (mm^2^)	2.17 ± 0.03	2.08 ± 0.06	2.23 ± 0.04	2.43 ± 0.14
Subendosteal area (mm^2^)	1.15 ± 0.02	1.11 ± 0.04	1.25 ± 0.04	1.20 ± 0.09

Data are means ± SEM for (n) mice.

^a^Significant effect of PTH, p<0.01.

^b^Significant effect of genotype, p<0.05.

**Fig 3:**
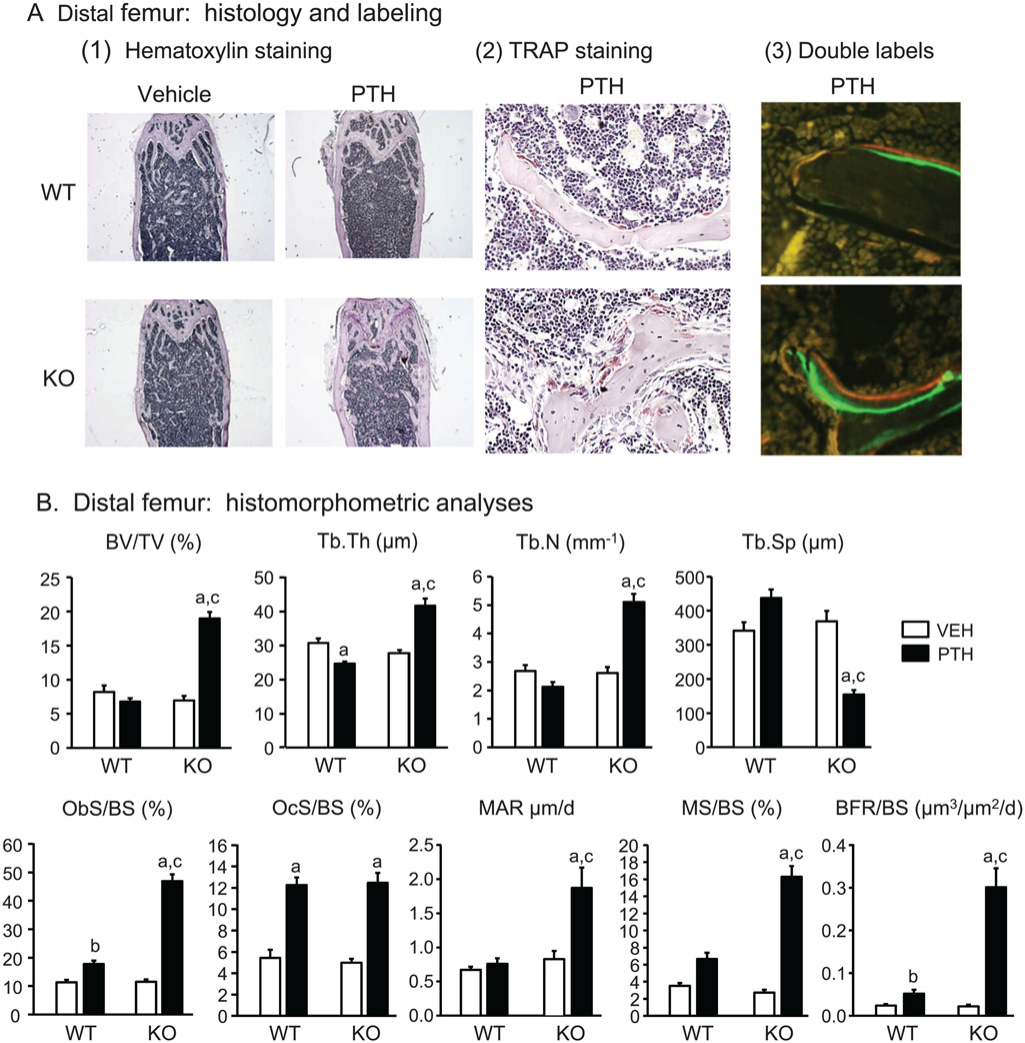
Static and dynamic histomorphometric analysis of distal femurs in mice infused for 12 days. **A.** Representative microscopic images of distal femur. (1) Hematoxylin staining at 20x magnification. (2) Tartrate resistant acid phosphatase (TRAP) staining and counter staining with hematoxylin at 400x magnification in PTH-infused WT and KO mice. (3) Double labeling with calcein (green) and demeclocycline (orange/brown) of trabeculae at 400x magnification in PTH-infused WT and KO mice. **B.** Histomorphometric analysis of distal femurs. Bars are means ± SEM for 7 WT and 7 KO mice treated with vehicle and 8 WT and 8 KO mice treated with PTH. ^a^Significant effect of PTH, p<0.01; ^b^p<0.05. ^c^Significant effect of genotype, p<0.01.

**Static and dynamic histomorphometry.** Histology supported the greater increase in trabecular bone mass by PTH in *Cox2* KO compared to WT mice (Fig. 3A-1). By static histomorphometry of the distal right femur (Fig. 3B), PTH increased BV/TV 2.7-fold in *Cox2* KO mice but decreased (not significantly) BV/TV 17% in WT mice, resulting in BV/TV being 2.8-fold greater in PTH-infused KO femurs compared to WT femurs. Tb.Th was increased 29% by PTH in KO mice and decreased 20% in WT mice. PTH increased Tb.N and decreased Tb.Sp in *Cox2* KO mice but not in WT mice. PTH caused a marked increase (4.0-fold) in % osteoblast surface (Ob.S/BS) in *Cox2* KO mice and a much smaller increase (1.6-fold) in WT mice (Fig. 3B). Osteoblasts were piled up around trabeculae in KO mice after PTH but not in WT mice (Fig. 3A-2). In contrast, PTH increased % osteoclast surface (Oc.S/BS) similarly, 2.3-fold and 2.5-fold, respectively, in both WT and KO femurs (Fig. 3B). Percent eroded surface was increased from 7.40 ± 0.68 to 16.6 ± 0.62 (p<0.01) in WT mice and from 8.86 ± 0.59 to 16.2 ± 0.88 (p<0.01) in KO mice by PTH, and there was no difference between the genotypes (data not shown). Dynamic histomorphometry (Fig. 3B) performed on the left femur showed increased MAR and MS/BS of 2.25-fold and 13.5-fold, respectively, with PTH in *Cox2* KO femurs, with little effect on WT femurs. BFR/BS was increased 13.5-fold by PTH in *Cox2* KO femurs and only 2.2-fold in WT femurs. These data indicate that PTH markedly increased osteoblast activity and number in *Cox2* KO mice with much less effect in WT mice.

**Fig 4:**
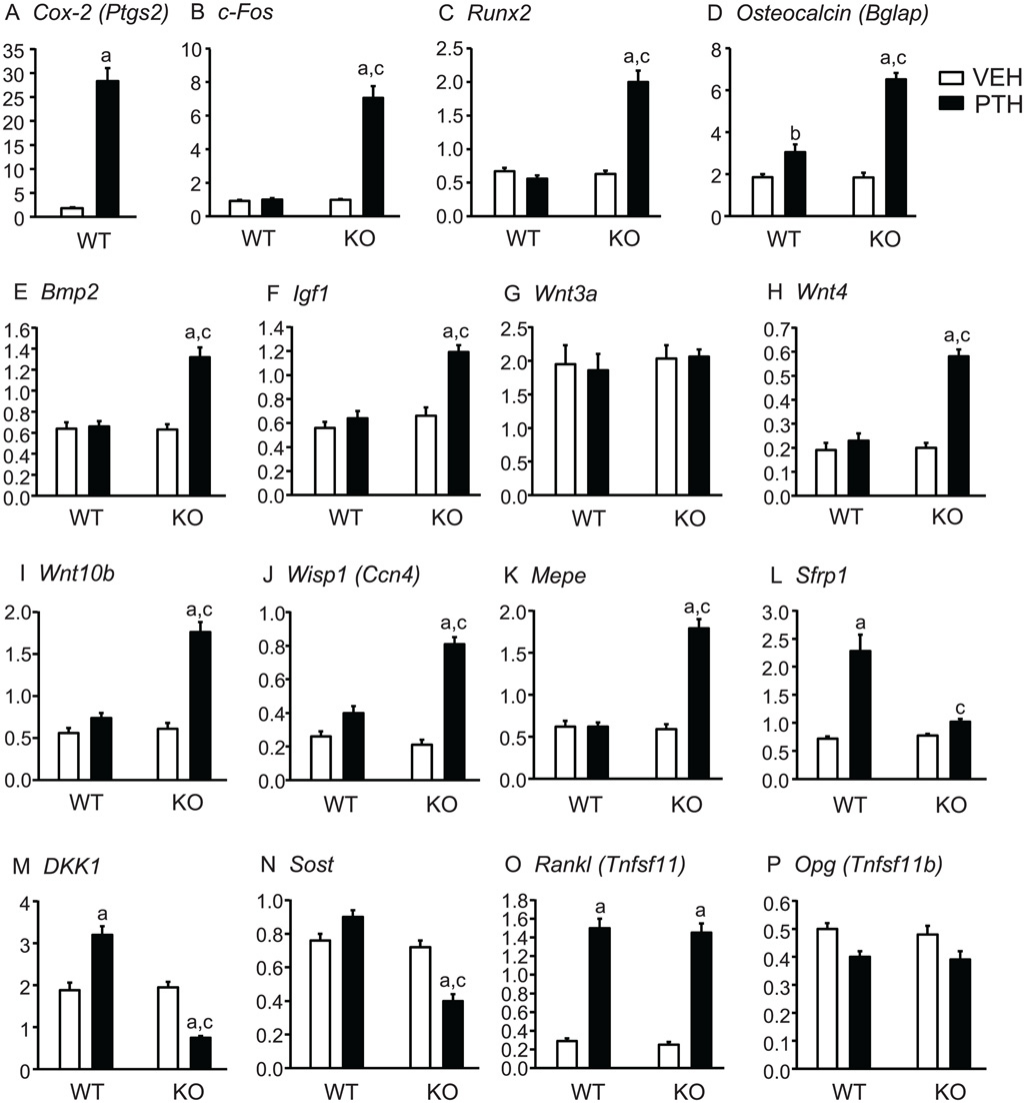
Tibial gene expression in mice infused with vehicle or PTH for 12 days. At the end of infusion, both tibiae from each mouse, minus ends, were combined for RNA extraction. mRNA expression was measured by qPCR, as described in Methods. Bars are means ± SEM for 7 WT and 7 KO mice infused with vehicle and 8 WT and 8 KO infused with PTH. ^a^Significant effect of PTH, p<0.01;^b^p<0.05. ^c^Significant effect of genotype, p<0.01.

**Tibial gene expression.** To confirm that PTH infusion could maintain continuously elevated *Cox2* expression, we measured *Cox2* mRNA in WT mice after the 12 days of infusion and found it to be 16-fold greater in PTH-treated compared to vehicle-treated mice (Fig. 4A). PTH stimulated an early response, cAMP-mediated gene, *c-Fos*, in KO mice but not in WT mice (Fig. 4B). Expression of *Runx2* and *Osteocalcin (Bglap)*, genes associated with early and late osteoblast differentiation, respectively, were markedly induced in KO tibiae by PTH with little or no effect in WT tibiae (Fig. 4C,D). Expression of genes for BMP-2, a potent anabolic agent [28], and IGF-1, one of several growth factors implicated in the anabolic effects of PTH [29] were increased by PTH only in *Cox2* KO mice (Fig. 4E,F).

PTH is thought to have anabolic effects via the Wnt signaling pathway. *Wnt3a* was not induced by PTH in either WT or KO mice (Fig. 4G). Expression of non-canonical *Wnt4*, proposed to mediate anabolic effects of PTH [2,30], was increased by PTH only in KO mice (Fig. 4H). *Wnt10b*, which has been shown to enhance osteoblast differentiation [31], was increased by PTH only in KO mice (Fig. 4I). Expression of *Wnt-inducible protein 1* (*Wisp1*), previously shown to be increased by PTH [32] and to positively influence osteogenesis [33], was also increased by PTH only in KO mice (Fig. 4J). *Mepe*, which may be a target for both Wnt and BMP-2 signaling [34], was also increased only in *Cox2* KO mice (Fig. 4K). In contrast to genes that may mediate Wnt signaling, expression of genes thought to inhibit Wnt signaling were lower in PTH-treated KO mice compared to PTH-treated WT mice. *Secreted frizzled-related protein1* (*Sfrp1)*, both the deletion and overexpression of which have been shown to blunt the anabolic effects of PTH, was increased by PTH in WT mice but not in KO mice (Fig. 4L). PTH decreased expression of the Wnt inhibitors, *Dkk1* and *Sost*, in *Cox2* KO mice but not in WT mice (Fig. 4M,N). Interestingly, PTH did not decrease *Sost* expression in WT mice.

As expected from the similar effects of PTH on serum CTX and OcS/BS in WT and KO mice, genes associated with resorption were regulated similarly by PTH. *Rankl* (*Tnfsf11*), which is critical for the formation and differentiation of osteoclasts, was markedly increased in both WT and KO mice (Fig. 4O) and *Opg* (*Tnfrsf11b*), the decoy receptor for Rankl that acts to inhibit resorption, was decreased (non-significantly) in both WT and KO mice (Fig. 4P). As a result, the ratio of *Rankl*/*Opg*, which determines resorption, was increased similarly in both WT and KO (data not shown).

### Study 2: PTH infusion for 21 days

To determine if longer PTH infusion would cause greater or lesser anabolic effects in *Cox2* KO mice, we infused mice for 3 weeks. Mice did not appear to be in distress nor did they lose weight despite the longer duration of infusion (Table 5). Similar to the shorter infusion, PTH increased serum calcium equally in WT mice and *Cox2* KO mice.

**Table 5:** Body weight and serum calcium (Ca) in 3-mo old wild type (WT) and *Cox2* KO mice infused with vehicle or PTH for 21 days.

Parameter	WT mice		*Cox2* KO mice	
	Vehicle (6)	PTH (7)	Vehicle (6)	PTH (7)
Start weight (g)	26.2 ± 0.31	26.1 ± 0.49	23.7 ± 0.23^b^	24.1 ± 0.38^b^
End weight (g)	28.9 ± 0.32	28.6 ± 0.48	26.4 ± 0.28^b^	27.2 ± 0.27^c^
Ca (mg/dl) at end	9.34 ± 0.18	11.3 ± 0.17^a^	9.24 ± 0.13	11.4 ± 0.13^a^

Data are means ± SEM for (n) mice.

^a^Significant effect of PTH, p<0.01.

^b^Significant effect of genotype, p<0.01,

^c^p<0.05.

**Fig 5:**
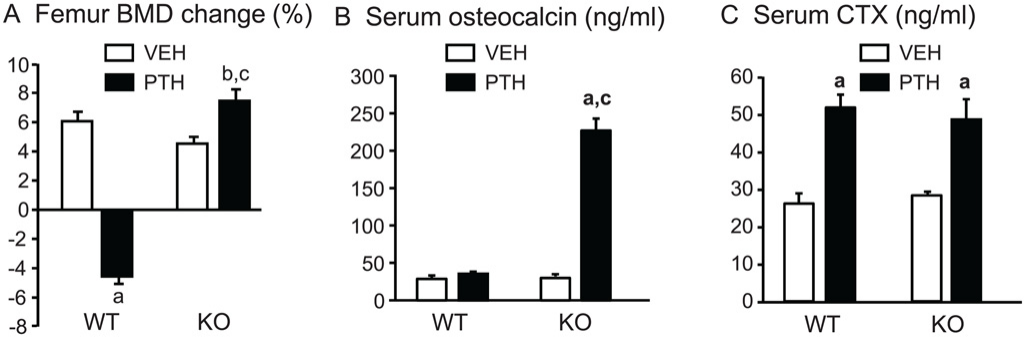
Femoral BMD and serum markers of bone turnover in mice infused for 21 days. **A.** BMD was measured *in vivo* at beginning and end of infusion with vehicle or PTH in WT and *Cox2* KO mice and the percent change calculated relative to the beginning BMD for each mouse. **B, C.** Serum parameters for bone formation (osteocalcin) and bone resorption (CTX) were measured at the end of study. Bars are means ± SEM for 6 WT and 6 KO mice infused with vehicle and 7 WT and 7 KO infused with PTH. ^a^Significant effect of PTH, p<0.01; ^b^p<0.05. ^c^Significant effect of genotype, p<0.01.

**Percent change in BMD and serum markers of turnover.** There was no significant difference between WT and *Cox2* KO femoral BMD at the beginning of the experiment (Fig. 5A). PTH infusion for 21 days converted the % change in femoral BMD in WT mice from a gain of 6.1% to a loss of 4.6%. In *Cox2* KO mice PTH increased the % change in BMD from 4.5% to 7.5%. These effects are similar to those seen with the shorter 12 day infusion. PTH increased serum osteocalcin, a marker of bone formation, 7.6-fold in KO mice but had no significant effect on osteocalcin in WT mice (Fig. 5B). PTH increased serum CTX similarly in WT (2.0-fold) and KO (1.7-fold) (Fig. 5C). Hence, as seen in the shorter infusion, the effects of PTH on formation, but not resorption, were enhanced in the *Cox2* KO mice compared to WT mice.

**Fig 6:**
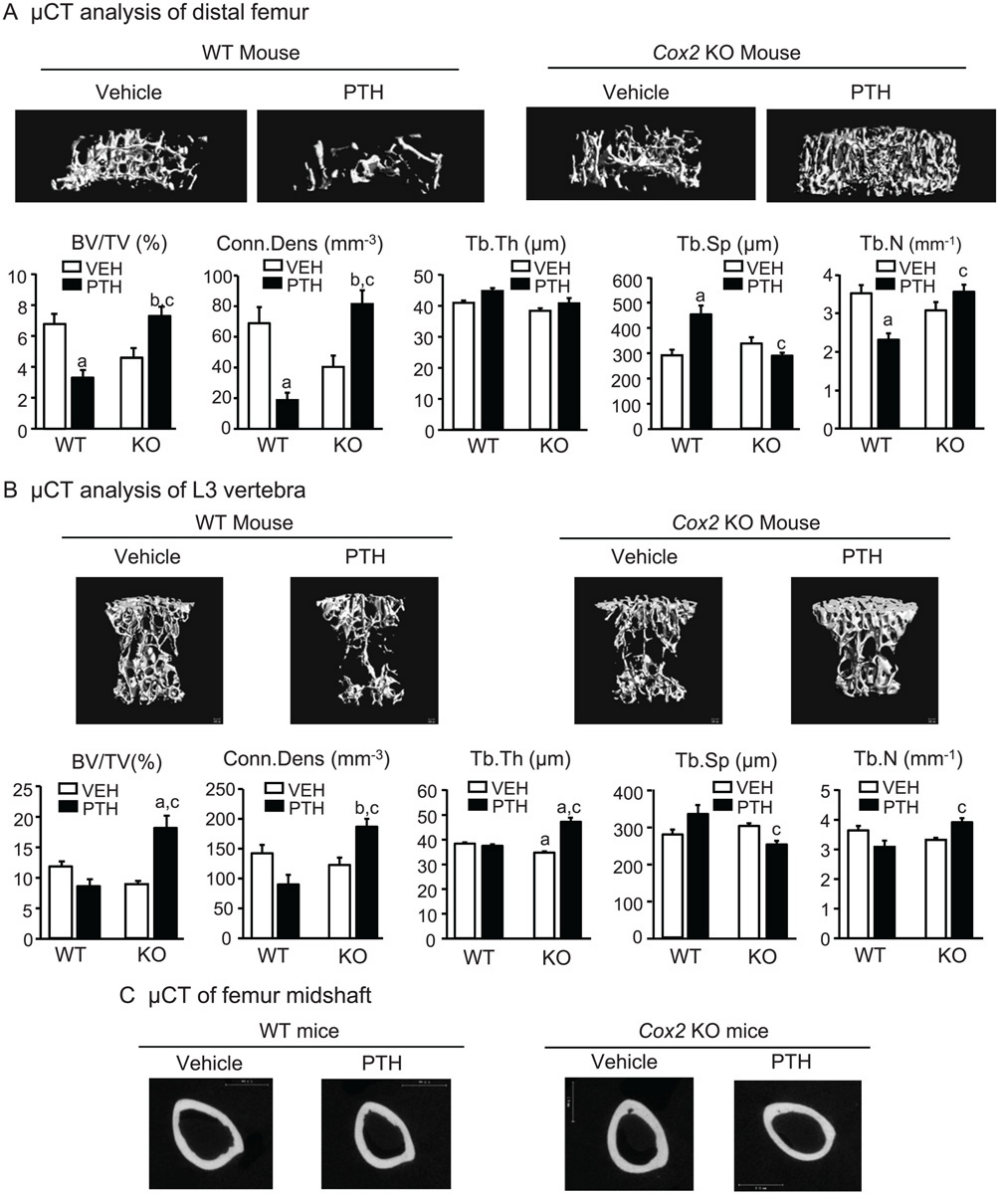
Morphometry of femoral trabecular bone and L3 vertebrae in mice infused for 21 days. **A.** Representative μCT images (top panel) and morphometric analyses (bottom panel) of trabecular bone in the metaphyseal region of distal femur. **B.** Representative μCT images (top panel) and morphometric analyses (bottom panel) of trabecular bone in L3 vertebrae. Bars are means ± SEM for 6 WT and 6 KO mice infused with vehicle and 7 WT and 7 KO mice infused with PTH. ^a^Significant effect of PTH, p<0.01; ^b^p<0.05. ^c^Significant effect of genotype, p<0.01. **C**. Representative μCT cross-sectional images of midshaft of femurs.

**μCT analyses of femur and vertebra.** PTH increased trabecular bone volume in femurs and L3 vertebrae of KO mice. In WT mice, PTH decreased femoral BV/TV 51% and this was associated with a 73% decrease in Conn.Dens, a 34% decrease in Tb.N and a 36% increase in Tb.Sp (Fig. 6A). In contrast, in *Cox2* KO mice, PTH increased BV/TV 52% and Conn.Dens 100%. As a result, BV/TV was 2.2-fold greater in KO compared to WT mice after PTH infusion and the Conn.Dens was 4.4-fold greater. Tb.Sp was 36% lower and Tb.N 52% greater in KO mice compared to WT mice after PTH. Tb.Th was not changed by PTH in either WT or KO. SMI was similar in vehicle-treated WT and KO mice (2.58 ± 0.04 and 2.82 ± 0.08, respectively) and unchanged in PTH-treated WT and KO mice (2.77 ± 0.06 and 2.87 ± 0.10, respectively).

μCT of the centrum of the L3 vertebrae showed significant increases in BV/TV of 91% with PTH infusion in *Cox2* KO mice (Fig. 6B). PTH increased Conn.Dens and Tb.Th in KO mice but not WT mice. Tb.Sp was decreased and Tb.N increased in KO mice relative to WT mice with PTH. There was no difference in SMI between vehicle- and PTH-treated mice (1.77 ± 0.10 and 2.03 ± 0.10, respectively). SMI was decreased by PTH (p<0.05) in KO mice (1.53 ± 0.16) compared to vehicle treatment (2.01 ± 0.07) and compared to PTH-treated WT mice.

PTH infusion increased cortical area in KO, but not WT, femurs at mid-diaphysis but unlike the 12 day infusion, cortical thickness was not increased (Fig. 6C, Table 6). Cortical porosity was doubled in the KO mice but because of the variability in measurements, the difference was not statistically significant.

**Table 6:** Cortical morphometry by μCT in the midshaft femoral region of WT and *Cox2* KO mice after infusion with vehicle or PTH for 21 days.

Parameter	WT mice		*Cox2* KO mice	
	Vehicle (6)	PTH (7)	Vehicle (6)	PTH (7)
Cortical area (mm^2^)	0.94 ± 0.02	0.91 ± 0.02	0.88 ± 0.03	1.05 ± 0.03^a^ ^,^ ^b^
Cortical thickness (mm)	0.21 ± 0.01	0.20 ± 0.01	0.20 ± 0.01	0.19 ± 0.01
Cortical porosity (%)	0.33 ± 0.06	0.31 ± 0.04	0.53 ± 0.11	1.05 ± 0.46
Subperiosteal area (mm^2^)	1.97 ± 0.05	2.12 ± 0.07	1.89 ± 0.04	1.92 ± 0.04
Subendosteal area (mm^2^)	1.03 ± 0.04	1.16 ± 0.06	1.01 ± 0.03	1.06 ± 0.03

Data are means ± SEM for (n) mice.

^a^Significant effect of PTH, p<0.01.

^b^Significant effect of genotype, p<0.05.

**Fig 7:**
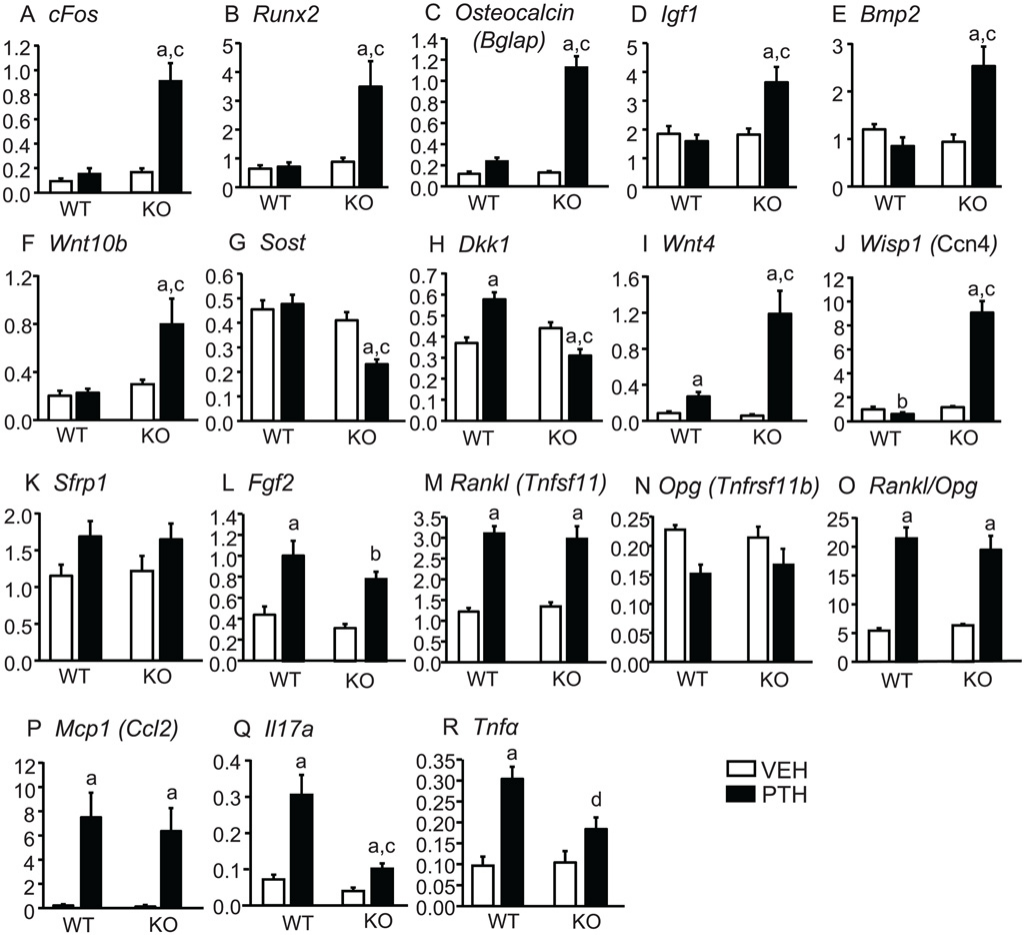
Tibial gene expression in mice infused with vehicle or PTH for 21 days. At the end of infusion, both tibiae were combined for RNA extraction and mRNA expression was measured by qPCR Bars are means ± SEM for 6 WT and 6 KO mice infused with vehicle and 7 WT and 7 KO infused with PTH. ^a^Significant effect of PTH, p<0.01; ^b^p<0.05. ^c^Significant effect of genotype, p<0.01; ^d^p<0.05.

**Tibial gene expression.** As in the 12 day infusion experiment, PTH increased *cFos;* genes associated positively with differentiation, *Runx2* and *Osteocalcin*; and genes for growth factors postulated to mediate some effects of PTH, *Igf1* and *Bmp2*, only in *Cox2* KO mice (Fig. 7A-E). PTH also increased *Wnt10b* (Fig. 7F) and *Wisp1* (Fig. 7J) only in KO mice and increased *Wnt4* expression to a much greater extent in KO than WT mice (Fig. 7I). Expression of the inhibitors of Wnt signaling, *Sost* and *Dkk1*, was decreased by PTH only in KO mice (Fig. 7G,H). Unlike the 12 day infusion, *Sfrp1* was regulated similarly in WT and KO mice (Fig. 7K). *Fgf2*, another growth factor reported to be important for effects PTH on bone [35], was regulated similarly by PTH in WT and *Cox2* KO mice (Fig. 7L).

Similar to the 12 day infusion, PTH regulated *Rankl* and *Opg* similarly in WT and KO mice and increased the ratio of *Rankl*/*Opg* equally in both WT and KO mice (Fig. 7M-O). *Monocyte chemotactic protein-1* (*Mcp-1* or *Ccl2*), proposed to enhance PTH-stimulated osteoclastogenesis by recruiting precursors [36], was increased similarly by PTH in WT and KO mice (Fig. 7P). Because the marrow was not flushed from tibiae before RNA extraction, we also examined expression of *Il17a* and *Tnfα*, which are proposed to mediate PTH effects on resorption and are likely to be highly expressed in T-cells [37]. PTH increased expression of both in WT and KO but levels were lower in KO mice (Fig. 7Q,R). It is possible that T-cells are also responsible for the PTH-stimulated *Wnt10b* seen in the tibiae [38].

## Discussion

In *Cox2* KO mice, PTH infusion was anabolic increasing femoral BMD, serum markers of bone formation, femoral and vertebral trabecular bone volume, cortical area, % osteoblast surface, bone formation rate, and expression of genes thought to reflect or mediate increased bone formation. In WT mice, on the other hand, the overall effect of PTH infusion was catabolic. PTH decreased femoral BMD in WT mice in both 12 and 21 day infusions and decreased BV/TV and Tb.N in all μCT analyses of femoral and vertebral bone from the 12 and 21 day infusions, although the decrease was statistically significant only in the femur in the 21 day infusion. Tb.Sp was significantly increased by PTH in three of the four analyses. However, PTH did stimulate small increases in serum P1NP, % osteoblast surface, bone formation rate, and *Osteocalcin* gene expression in the 12 day infusion. Otherwise, there was little or no effect on expression of genes thought to mediate PTH anabolic effects in WT mice. These results are similar to those seen in other studies of continuous PTH in WT mice [32,39], despite marked differences in PTH dose, mouse age, gender or background, and are consistent with PTH stimulating both resorption and formation but stimulating resorption more than formation because formation is suppressed.

In contrast to the differences between WT and KO mice in PTH-stimulated anabolic effects, serum markers of bone resorption, % osteoclast surface, and expression of genes associated with increased osteoclastic differentiation, such as *Opg* and *Rankl*, were regulated similarly by PTH in both WT and KO mice. Thus, the absence of *Cox2* enhanced the anabolic effects of continuous PTH infusion without changing the effects on resorption. Loss of bone mass with prolonged elevation of PTH has usually been attributed to resorption being increased more by PTH than formation [40,41]. Our results suggest that trabecular bone loss in WT mice in response to continuous PTH infusion is not due to increased resorption but to suppression of PTH-stimulated bone formation and, specifically, that this suppression is due to Cox2 expression.

Femoral and vertebral BV/TV measured by μCT were increased 127% and 95%, respectively, in PTH-infused *Cox2* KO mice compared to vehicle-infused KO mice after 12 days of infusion and 52% and 91%, respectively, after 21 days. As expected if resorption > formation, loss of trabecular bone volume in WT mice tended to increase with the longer infusion. Femoral and vertebral BV/TV were decreased 20% (non-significant) and 15% (non-significant), respectively, in PTH-infused compared to vehicle-infused WT mice after 12 days of infusion and 51% (significant) and 28% (non-significant), respectively, after 21 days. Thus, in the absence of *Cox2* the anabolic effects of PTH infusion were maintained but there was no advantage of the longer infusion.

Suppression of the anabolic response to continuous PTH was predicted by our *in vitro* studies [23]. Using bone marrow stromal cell cultures from WT and *Cox2* KO mice, continuous PTH stimulated osteoblastic differentiation only if Cox2 expression or activity was absent or if osteoclast formation was blocked. *In vitro*, we found that bone marrow macrophages (BMMs), committed to become osteoclasts, could secrete a factor that acted on osteoblasts to block PTH-stimulated osteoblastic differentiation. Secretion of this inhibitory factor required the presence of PGE_2_, which was produced by the PTH induction of *Cox2* in osteoblastic cells and/or by BMMs exposed to Rankl. We hypothesized that the *in vivo* PTH infusion model might reproduce our *in vitro* effects because elevated levels of PTH and PGE_2_ levels might be sustained continuously. Indeed, markedly elevated *Cox2* expression and serum PGE_2_ in WT mice were still evident at the end of the 12 day infusion. In vehicle-treated WT mice, serum PGE_2_ was detectable. However, despite the constitutive expression of Cox1, we could not detect PGE_2_ in the serum of *Cox2* KO mice, with or without PTH. These results suggest that the PGE_2_ produced by *Cox1* is not sufficient to compensate for the absence of *Cox2*.

This study raises questions about longstanding expectations for effects of PGE_2_ on bone. PGE_2_ can be a potent stimulator of resorption and is induced by multiple resorption agonists [17,42,43]. It is, therefore, surprising to find little or no effect of absent *Cox2* on PTH-stimulated resorption. PGE_2_ can also have anabolic effects on bone [13,18,19]. In our *in vitro* studies, the inhibitory factor secreted by early osteoclasts in response to PGE_2_ had no effect on the osteogenic actions of PGE_2_ alone but did inhibit the osteogenic actions of both PGE_2_ and PTH when they were given together [23]. It is possible that something similar occurs *in vivo*. Attempts to establish anabolic murine models of systemically applied PGE_2_ may have been limited by the effects of PGE_2_ to stimulate secretion of this inhibitory factor, which would then block the anabolic effects of both endogenous PTH and exogenous PGE_2_.

PTH is likely to have its anabolic actions mediated via cAMP/ PKA signaling [44]. There are multiple pathways by which PTH-stimulated cAMP/ PKA signaling can engage Wnt signaling [45,46]. Our gene expression studies suggest that many cAMP-mediated genes are significantly stimulated by PTH only in the *Cox2* KO mice. On the other hand, some genes that were increased similarly in the WT and *Cox2* KO mice by PTH, such as *Rankl*, are also reported to be regulated by PTH via cAMP/PKA [47]. This lack of differential response of Rankl to PTH in WT and KO mice could be due to PTH regulation via non-cAMP signaling or to actions of PTH on cell types that do not have the receptor(s) for the inhibitor. Osteocytes, which may be responsible for much of PTH-stimulated Rankl production [48,49], also express *Sost* and *Mepe*, which were differentially regulated by PTH in WT and KO mice. Alternatively, T lymphocytes may secrete Rankl in response to PTH, as well as cytokines that can stimulate Rankl in osteoblasts, such as Il17a and Tnfα [37]. PTH infusion increased expression of both *Il17a* and *Tnfα* in WT and *Cox2* KO mice, and although levels were lower in KO mice, they were still significantly elevated. It is possible that the anabolic and catabolic effects of PTH are mediated by different cells types.

Our findings in this *in vivo* study, in conjunction with our *in vitro* studies [23], suggest a novel role for Cox2 produced PGs to suppress PTH-stimulated osteogenic or anabolic activity. The importance of this finding for humans is likely to be in situations where either PTH or Cox2 expression is substantially elevated and, because our *in vitro* data indicate that the inhibitory factor can be secreted by early osteoclasts [23], also in situations where osteoclastogenesis is stimulated. *Cox2* KO mice have mildly elevated PTH and slightly lower bone mass than WT mice [21,26], suggesting that loss of *Cox2* does not increase the anabolic effects of PTH under these conditions. Cox2 may act to decrease the anabolic actions of PTH in chronic inflammatory conditions where both Cox2 activity and osteoclast number are elevated. In this regard, in the Canadian Multicentre Osteoporosis Study, Cox2 inhibitor use was associated with a higher BMD in postmenopausal women not using estrogen replacement therapy and perhaps, therefore, with a higher cytokine milieu [50]. Our findings may also be important for treatment with intermittent PTH since the anabolic effects of intermittent PTH injections were greater in *Cox2* KO mice than in WT mice [21]. It is possible that improved therapeutic protocols for delivering PTH to treat osteoporosis could be designed employing short periods of Cox2 inhibition.
